# The differences in homeostasis model assessment values in type 2 diabetic patients with different lengths of history of diabetes

**DOI:** 10.20945/2359-3997000000134

**Published:** 2019-04-26

**Authors:** Chen Wang, Zaibo Liu, Peng Zhang, Xiaolong Ma, Kui Che, Yangang Wang

**Affiliations:** 1 Endocrinology Department The Affiliated Hospital of Qingdao University Qingdao Shandong China Endocrinology Department, The Affiliated Hospital of Qingdao University, Qingdao, Shandong, China; 2 Department of General Surgery People’s Hospital of Haiyang Yantai Shandong China Department of General Surgery, People’s Hospital of Haiyang, Yantai, Shandong, China; 3 Department of Gastroenterology, The Affiliated Hospital of Qingdao University Qingdao Shandong China Department of Gastroenterology, The Affiliated Hospital of Qingdao University, Qingdao, Shandong, China; 4 Laboratory of Thyroid Disease The Affiliated Hospital of Qingdao University Qingdao Shandong China Laboratory of Thyroid Disease, The Affiliated Hospital of Qingdao University, Qingdao, Shandong, China

**Keywords:** Type 2 diabetes, islet function, insulin resistance, history of disease

## Abstract

**Objective:**

Type 2 diabetes (T2DM) is characterized by the progressive deterioration of pancreatic islet β-cell function over time and insulin resistance. Knowing more about the differences in pancreatic islet function in T2DM patients who have had diabetes for different lengths of time can help improve therapy for T2DM.

**Subjects and methods:**

We conducted a cross-sectional study to compare islet β-cell function and insulin resistance in T2DM patients (n = 3,254) who had had diabetes for different lengths of time and those in normal controls (n = 794) using ANOVA and LSD analysis.

**Results:**

We found that compared with that in normal controls, HOMA-β in T2DM patients with a history of diabetes of less than 1 year was lower (approximately 52% of that of normal controls, p = 0.003), while HOMA-IR in these patients was higher (approximately 50% of that of normal controls, p = 0.007). Compared with that in other diabetic patients, HOMA-β in patients with a history of diabetes of more than 30 years was the lowest. HOMA-IR in patients with a history of diabetes of between 20 and 30 years was lower than that in other diabetic patients (p < 0.05).

**Conclusions:**

There were obvious decreases in HOMA-β and increases in HOMA-IR in T2DM patients with a history of diabetes of less than 1 year compared with those in normal controls. Therefore, early screening and intervention for T2DM might help improve islet function and delay the progression of diabetes.

## INTRODUCTION

Type 2 diabetes (T2DM) is a major global health issue with an incidence of more than 9.7% in China (1). According to a report from the American Diabetes Association (ADA, Philadelphia, PA, USA), the total number of Americans living with diabetes will increase 64% by 2025 (2). Moreover, diabetic complications, such as kidney failure and cardiovascular diseases, can decrease the quality of life and limit the regular activity and productivity of individuals with T2DM (3).

T2DM is characterized by the progressive deterioration of islet β-cell function over time and insulin resistance. Initially, islet β cells increase insulin secretion to meet insulin demand, but gradually they cannot compensate for insulin resistance, and hypoinsulinism and hyperglycemia appear (4). Autopsy studies demonstrated a 63% reduction in the islet mass of T2DM patients compared to the islet mass of matched normal controls (5). Subjects with impaired fasting glucose (IFG) were shown to have a reduction of 40% of relative β-cell volume, suggesting that loss of β-cell mass was present at early stages of T2DM (5). It was also reported that β-cell mass was already reduced by 50% at the time of diagnosis of T2DM and that it continued to decline throughout the course of T2DM (6). The reduced β-cell mass was not due to reduced formation of islets or regeneration but was caused by increased rates of apoptosis in islets. The decline in β-cell function in T2DM drives the progressive deterioration of glycemic control and increases the requirement for insulin (7).

However, there have been few studies on the decline of islet β-cell function in T2DM patients with a long history of the disease. In the present study, we conducted a cross-sectional study to compare islet ‘β-cell function and insulin resistance in T2DM patients who had had the disease for different lengths of time and to observe the patterns of insulin secretion and insulin resistance as the length of history of having diabetes changed.

## SUBJECTS AND METHODS

### Study design

The study was an open, single-center, cross-sectional study. Participants were involved according to the time of diagnosis of T2DM.

### Participants

The study protocol was approved by the Ethical Committee of the Affiliated Hospital of Qingdao University. Informed consent in accordance with the Declaration of Helsinki was provided by every patient. T2DM patients who were in the Department of Endocrinology at the Affiliated Hospital of Qingdao University from 2013-2016 were enrolled in the present study. Patients with T1DM, ketoacidosis, hyperosmolar state, and severe acute disease were excluded. According to their medical history, patients were divided into 5 groups: group I (history of T2DM of less than 1 year), group II (history of T2DM between 1 and 10 years), group III (history of T2DM between 10 and 20 years), group IV (history of T2DM between 20 and 30 years), and group V (history of T2DM of more than 30 years). Healthy people with normal glucose who were age- and sex-matched were used as normal controls.

### Examinations

All medical histories were recorded by two doctors. Height, weight, waist and hip circumference, body mass index (BMI), blood glucose, fasting C peptide, liver function, and hemoglobin were measured. Blood glucose, fasting C peptide, liver function and hemoglobin were measured by hexokinase and chemiluminescence methods. Islet function was evaluated with the modified homeostasis model assessment for β-cell function (HOMA-β), which was calculated using fasting C peptide level (pmol/L) and fasting blood glucose (FPG, mmol/L), and insulin resistance (IR) was evaluated using the modified homeostasis model assessment for insulin resistance (HOMA-IR). The formulas were as follows: HOMA-β = 20 * fasting C peptide / (FPG - 3.5) and HOMA-IR = fasting C peptide * FPG / 22.5 (8,9).

### Statistical analysis

All data were analyzed by Empowerstats statistical software, and the results are presented as x ± S. The differences between groups were analyzed with ANOVA. Multiple comparisons were performed by LSD analysis. A two-sided *p* < 0.05 was considered statistically significant.

## RESULTS

### The characteristics of the patients

A total of 3254 patients with T2DM and 794 normal controls were enrolled in this study. The clinical characteristics of all participants are shown in Table 1.

**Table 1:** The clinical characteristics of all participants

	Normal controls	group I	group II	group III	group IV	group V
Number	794	568	924	978	532	252
Age	58.4 ± 11.6	55.8 ± 13.2	56.4 ± 12.4	58.7 ± 15.3	59.8 ± 15.2	61.3 ± 13.4
History of T2DM	–	0.5 ± 0.2	5.0 ± 2.4	13.2 ± 2.9	22.1 ± 2.9	31.1 ± 2.5
Weight (kg)	73.1 ± 12.9	73.8 ± 11.6	73.2 ± 12.4	72.3 ± 12.3	72.1 ± 11.6	69.3 ± 13.0
BMI (kg/m^2^)	27.0 ± 3.3	26.1 ± 4.1	26.0 ± 3.7	26.5 ± 4.7	26.6 ± 3.7	25.7 ± 3.4
SBP (mmHg)	138.9 ± 80.6	132.0 ± 17.4	137.1 ± 19.4	140.0 ± 19.4	142.3 ± 20.0	142.3 ± 19.8
DBP (mmHg)	87.6 ± 12.7	81.6 ± 10.4	82.5 ± 11.3	80.4 ± 11.0	77.1 ± 11.2	75.7 ± 9.6
HbA1c (%)	5.4 ± 0.4	9.7 ± 2.4*	8.6 ± 2.1*	8.7 ± 1.9*	8.9 ± 1.8*	8.9 ± 1.6*
FBG (mmol/l)	5.3 ± 0.4	7.8 ± 2.5*	8.0 ± 2.6*	7.9 ± 2.9*	7.8 ± 3.0*	7.9 ± 2.6*

* *p* < 0.05, compared with normal controls. Levels of HbA1c and FBG in the diabetic group were higher than those in the normal controls.

BMI: body mass index; SBP: systolic blood pressure; DBP: diastolic blood pressure; HbA1c: glycated hemoglobin A1c; FBG: fasting blood glucose.

### The differences in levels of fasting C peptide, HOMA-β and HOMA-IR in patients with different histories of T2DM

**Figure 1:**
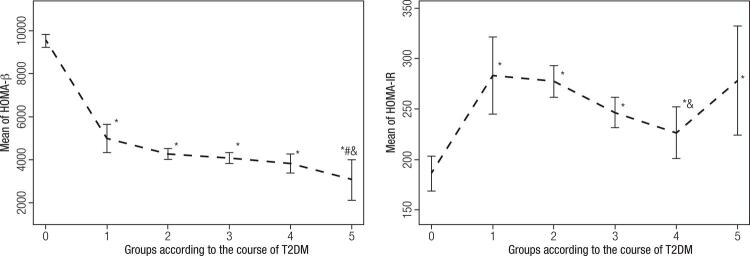
Differences in the levels of HOMA-β and HOMA-IR as a function of length of history of T2DM. Compared with levels in normal controls, HOMA-β in T2DM patients with a history of diabetes of less than 1 year decreased approximately 48% (*p* = 0.003), while HOMA-IR increased approximately 50% (*p* = 0.007). Compared with levels in other diabetic patients, HOMA-β in patients with a history of diabetes of more than 30 years was the lowest. HOMA-IR in patients with a history of diabetes of between 20-30 years was lower than that in other diabetic patients. * Compared with normal controls, *p* < 0.01; # compared with T2DM patients with a history of diabetes of less than 1 year, *P* < 0.05; & compared with T2DM patients with a history of diabetes of between 10 and 20 years, *p* < 0.05.

As shown in Table 2, HOMA-β in T2DM patients with a history of diabetes of less than 1 year was approximately 52% of that of normal controls (4973.8 vs 9553.7, *p* = 0.003), although there was no difference in the levels of fasting C peptide between T2DM patients with a history of diabetes of less than 1 year and that of normal controls. As shown in Figure 1, HOMA-β decreased with the development of T2DM. For T2DM patients with a history of diabetes of more than 30 years, there was only approximately 32% of pancreatic islet function left compared to the function in normal controls (3067.0 vs 9533.7, as shown in Table 2). Compared with that in other diabetic patients, HOMA-β in patients with a history of more than 30 years of diabetes was the lowest. HOMA-IR in T2DM patients with a history of diabetes of less than 1 year was higher (approximately 50%) compared to that of normal controls (283.0 vs 185.8, *p* = 0.007). HOMA-IR in patients with a history of between 20-30 years of diabetes was lower than that of other diabetic patients (*p* < 0.05). These data were analyzed using Empowerstats statistical software for overall HOMA-β and HOMA-IR, correlation of HOMA-β and HOMA-IR with the history of diabetes, and HOMA-β and HOMA-IR in each group as shown in Figure 1.

**Table 2:** The differences in levels of fasting C peptide, HOMA-β and HOMA-IR in patients with different lengths of history of T2DM

	Normal controls	group I	group II	group III	group IV	group V
Fasting C peptide (ng/mL)	2.4 ± 0.8	2.3 ± 1.3	2.2 ± 1.2*	2.0 ± 1.1*^&^	1.9 ± 1.5*^#&^	1.9 ± 1.2*^#&^
HOMA-β	9533.7 ± 4276.8	4973.8 ± 4144.9*	4268.7 ± 2851.8*	4096.4 ± 3155.7*	3826.2 ± 3289.6*	3067.0 ± 1434.6^*#&^
HOMA-IR	185.8 ± 64.8	283.0 ± 191.5*	277.5 ± 251.5*	246.4 ± 193.4*	226.2 ± 212.6*^&^	278.0 ± 321.9*

* Compared with normal controls, *P* < 0.05; ^#^ compared with T2DM patients with a history of disease of less than 1 year, *p* < 0.05; ^&^ compared with T2DM patients with a history of disease between 10 and 20 years, *p* < 0.05.

### Differences in the levels of fasting C peptide, HOMA-β and HOMA-IR in T2DM patients with different BMIs

**Figure 2:**
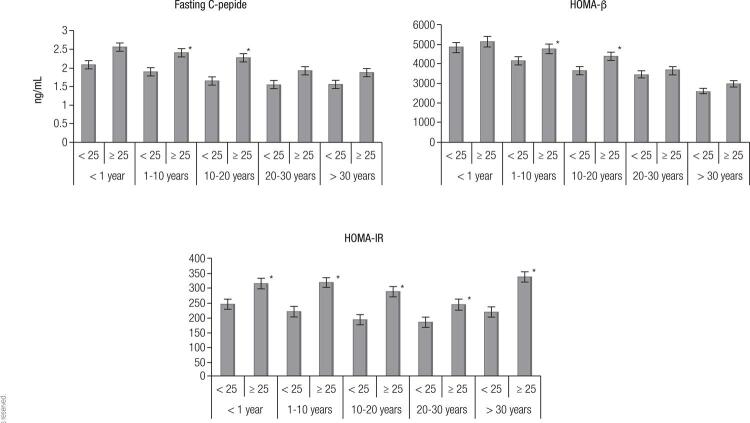
Differences in the levels of fasting C peptide, HOMA-β and HOMA-IR in patients with different BMIs.

We further analyzed differences in the levels of fasting C peptide, HOMA-β and HOMA-IR in T2DM patients with different BMIs. A BMI = 25 kg/m^2^ was set as the cut-off point. As shown in Figure 2, in group II (history of diabetes between 1-10 years) and group III (history of diabetes between 10-20 years), levels of fasting C peptide and HOMA-β in patients with BMI ≥ 25 kg/m^2^ were significantly higher than those in patients with BMI < 25 kg/m^2^ (*p* < 0.05). There were no significant differences in the levels of fasting C peptide and HOMA-β in patients with different BMIs in group I (history of diabetes < 1 year), group IV (history of diabetes between 20-30 years) and group V (history of diabetes of more than 30 years). For all T2DM patients, regardless of the history of diabetes, the levels of HOMA-IR in patients with BMI ≥ 25 kg/m^2^ were significantly higher than those in patients with BMI < 25 kg/m^2^ (*p* < 0.05).

In group II (history of diabetes between 1 and 10 years) and group III (history of diabetes of between 10 and 20 years), the levels of fasting C peptide and HOMA-β in patients with BMI ≥ 25 kg/m^2^ were significantly higher than those in patients with BMI < 25 kg/m^2^ (*p < 0.05). For all T2DM patients, the levels of HOMA-IR in patients with BMI ≥ 25 kg/m^2^ were significantly higher than those in patients with BMI < 25 kg/m^2^ (*p < 0.05).

## DISCUSSION

Pancreatic β-cells play a critical role in the development of T2DM. With the development of T2DM and an increase in age, both β-cell integrity and function declined and were accompanied by a decline in the ability of β-cells to be restored (10-12). In the present study, we investigated the difference in pancreatic islet secretion function in T2DM patients who have had the disease for different lengths of time by a cross-sectional study and the patterns of insulin secretion and insulin resistance as the length of time of having diabetes changed. We found that HOMA-β in T2DM patients with a history of diabetes of less than 1 year was approximately 52% that of normal controls, and HOMA-β in T2DM patients with a history of more than 30 years of diabetes was only approximately 32% of the pancreatic islet function left in normal controls. HOMA-IR in T2DM patients with a history of diabetes of less than 1 year was 1.5-fold that of normal controls, while HOMA-IR in patients with a history of diabetes of between 20-30 years was lower than that in other diabetic patients.

It was accepted that hyperglycemia and lipotoxicity played roles in the possible mechanisms underlying impaired β-cell function (13,14). Exposure of β-cells to hyperglycemia might promote efflux of insulin secretory granules from the β-cell, leaving less insulin available for release in response to further hyperglycemia (15,16). In addition, it was suggested that increased glucose levels could activate the hexosamine pathway, resulting in excess generation of reactive oxygen species and the inhibition of insulin gene transcription and insulin secretion (17,18). Accumulated fatty acids and their metabolic products might have a negative effect on the conversion of proinsulin to insulin and decrease nitric oxide production, inhibiting glucose oxidation and inducing β-cell apoptosis (19-21).

Additionally, we found that in patients with a history of diabetes of between 1-20 years, the levels of fasting C peptide and HOMA-β in patients with a BMI ≥ 25 kg/m^2^ were significantly higher than those in patients with a BMI < 25 kg/m^2^. Levels of HOMA-IR in patients with BMI ≥ 25 kg/m^2^ were significantly higher than those in patients with BMI < 25 kg/m^2^. It was reported that β-cell mass decreased by approximately 40 and 65% in lean and obese individuals with T2DM, respectively, compared to the cell mass of age- and BMI-matched nondiabetic individuals. In approximately two-thirds of obese individuals who did not develop T2DM, pancreatic islet mass increased to compensate for increased insulin demand (22,23). Changes in β-cell mass might occur several years before hyperglycemia appears, as suggested by the United Kingdom Prospective Diabetes Study (UKPDS) (24). Reduction in β-cell mass might occur following β-cell apoptosis and reduced generation of new cells (25,26).

Since a great proportion of patients with diabetes did not know about their disease for many years before the diagnosis, a possible limitation of the present study was the variable about disease history. A larger sample might reduce this bias. As T2DM patients with normal weight are quite different from those with BMI > 25 kg/m^2^, a further detailed investigation of islet function in T2DM patients with normal weight will be performed.

In the present study, we found that compared with that in normal controls, HOMA-β in T2DM patients with a history of diabetes of less than 1 year was lower (approximately 52% that of normal controls), while HOMA-IR was higher (approximately 50% that of normal controls). With the development of diabetes, HOMA-β gradually decreased, while there was no significant difference in HOMA-IR over time. These results could allow us to better understand T2DM and its development, thereby individualizing treatment for T2DM patients with different lengths of history of diabetes according to their condition and islet function.
